# Supplementary material for: A Single-Cell Transcriptomic Analysis of the Mouse Hippocampus After Voluntary Exercise
Source: Mol Neurobiol. 2024 Jan 13;61(8):5628–45. doi: 10.1007/s12035-023-03869-9 (PMC11249425; doi:10.1007/s12035-023-03869-9)
Supplement: Supplementary file 1 — Supplementary file1 (PDF 63937 KB) [file 12035_2023_3869_MOESM1_ESM.pdf]

Supplementary figures 1- 8

Supplementary figure 1

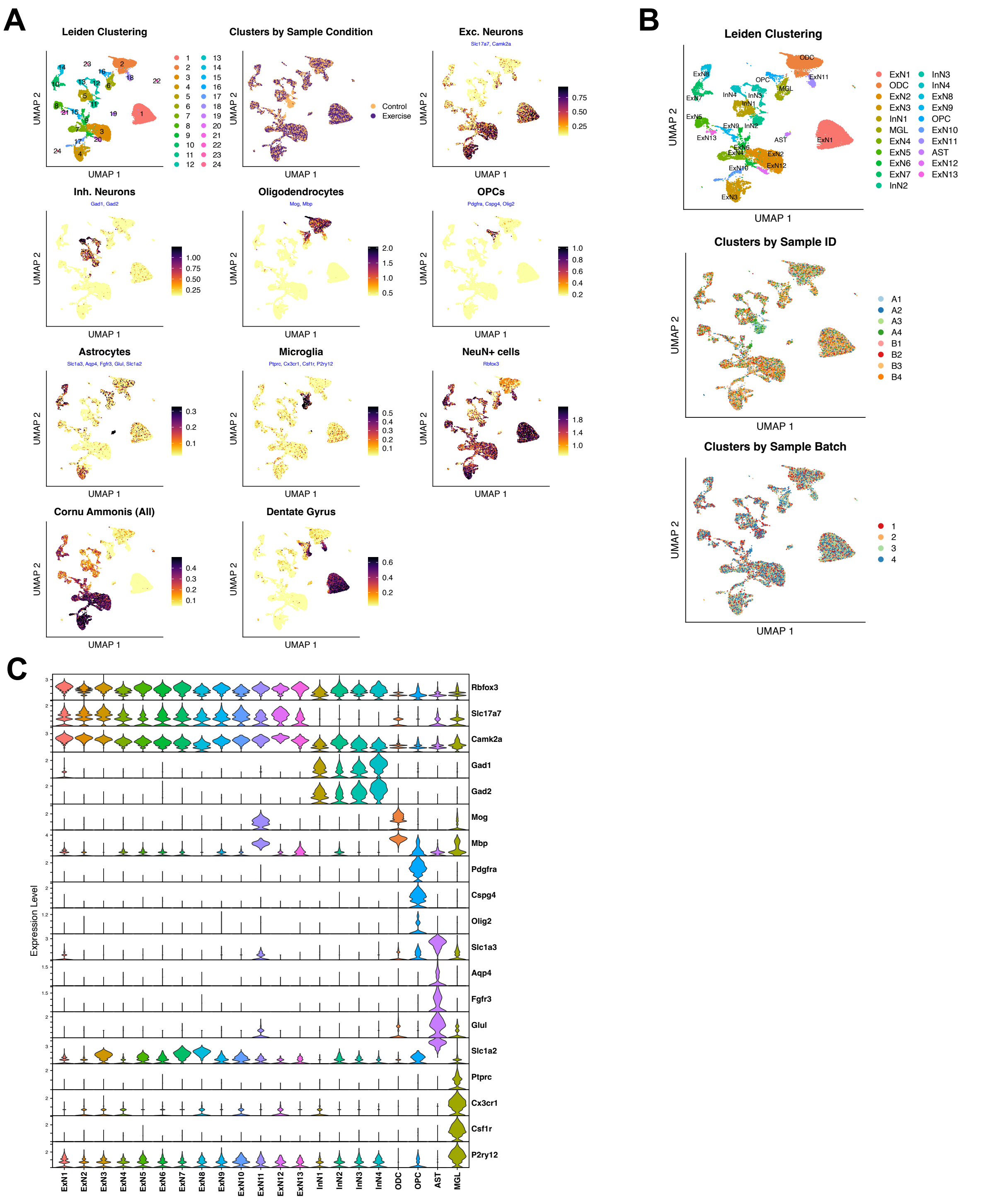

Supp. Fig. 1: Cluster annotation. A. UMAP plots with originally identified (unannotated) cell clusters colored by average module score (expression) of marker genes specific to the different cell types/hippocampal regions. <u>It is important to note that 2-5 marker genes are used to classify different cell types, while the classification of the CA and DG regions is based on 27 and 38 marker genes, respectively (see also Supp. Table 2). Especially the InN2 cluster,</u>

which will be discussed later in our manuscript as a cluster that may reflect a specific state of adult neurogenesis, appears to show some expression of CA-specific genes. However, the mean expression value of CA genes for the InN2 cluster is 0.14 when compared to clusters ExN2, 3, 4, 6, and 10, 12 (ranging from 0.33 to 0.46), which more clearly classify as CA. Thus, the fact that the InN2 cluster expresses some CA genes would not suggest that adult neurogenesis occurs in the CA region but rather indicate that the CA and DG marker gene lists are not suitable to classify the InN2 cluster. Please also see the violin plots in panel C. **B.** UMAP plots with cell clusters colored by annotated cell-type *(top)*, sample (mouse) of origin *(middle)* and sequencing batch *(bottom)*. **C.** Violin plots showing normalized expression of individual marker genes specific to the different cell types, after cell-type specific annotation of clusters *(ExN1-13: excitatory neurons; InN1-4: inhibitory neurons; ODC: oligodendrocytes; OPC: oligodendrocyte precursor cells; AST: astrocytes; MGL: microglia).*

Supplementary figure 2

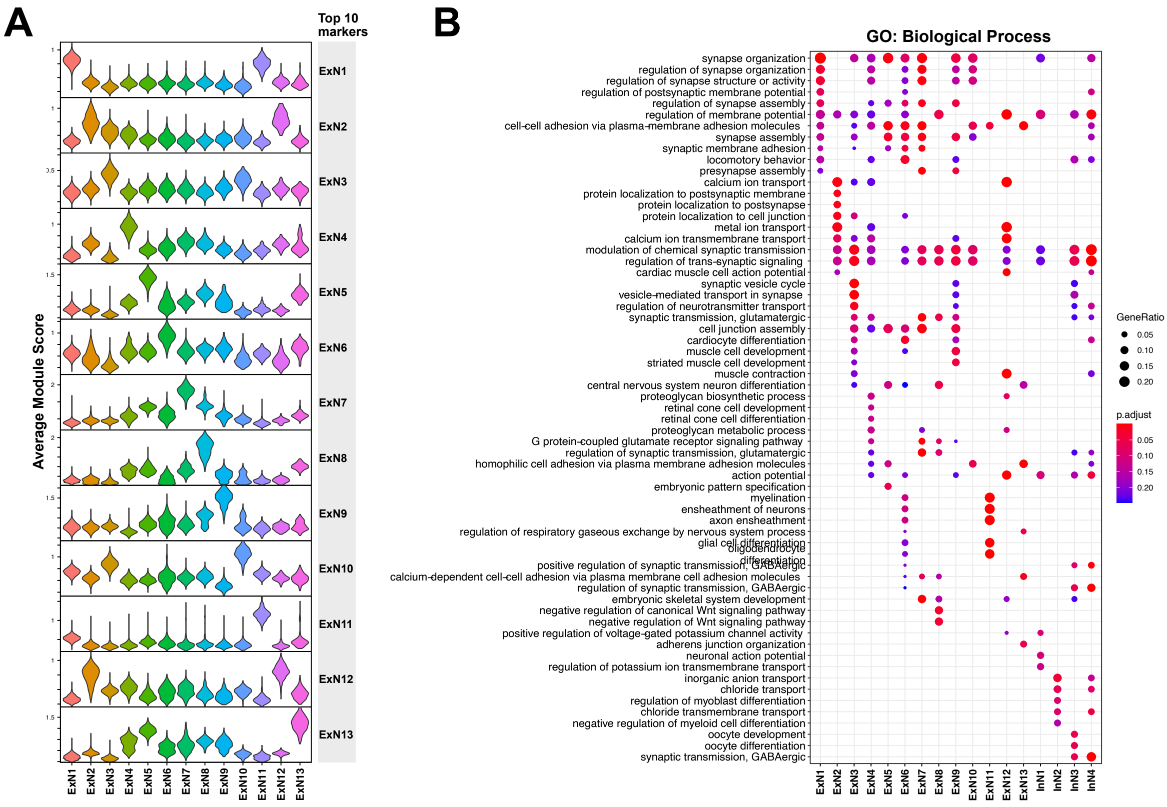

Supp. Fig. 2: A. Violin plots showing average module score (expression) of the top 10 computationally detected marker genes specific to each of the 13 different excitatory neuron clusters (ExN1-13). **B.** Dotplot showing the top 5 significant GO biological process terms enriched among the top 50 marker genes of the excitatory and inhibitory neuron clusters.

Supplementary figure 3

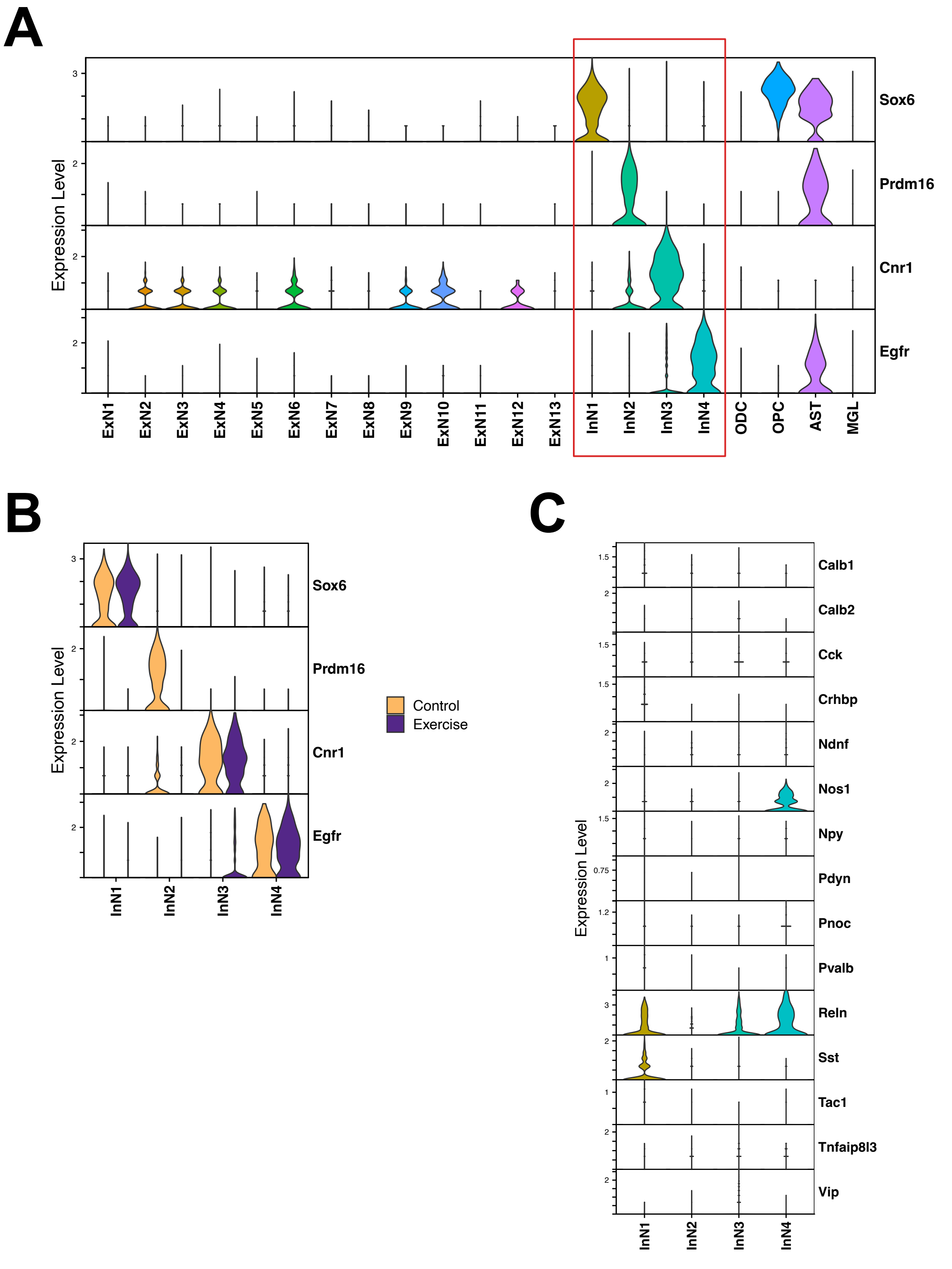

Supp. Fig. 3: A. Violin plots showing normalized expression of the top marker gene specific to each inhibitory neuron cluster in all cell-types *(ExN1-13: excitatory neurons; InN1-4: inhibitory neurons; ODC: oligodendrocytes; OPC: oligodendrocyte precursor cells; AST:*

astrocytes; MGL: microglia). **B.** Violin plots showing normalized expression of the top marker gene specific to each inhibitory neuron cluster (as above), in only the inhibitory clusters, further split by experimental condition. **C.** Violin plots showing normalized expression of gene markers used for canonical classification of inhibitory neuron subtypes.

Supplementary figure 4

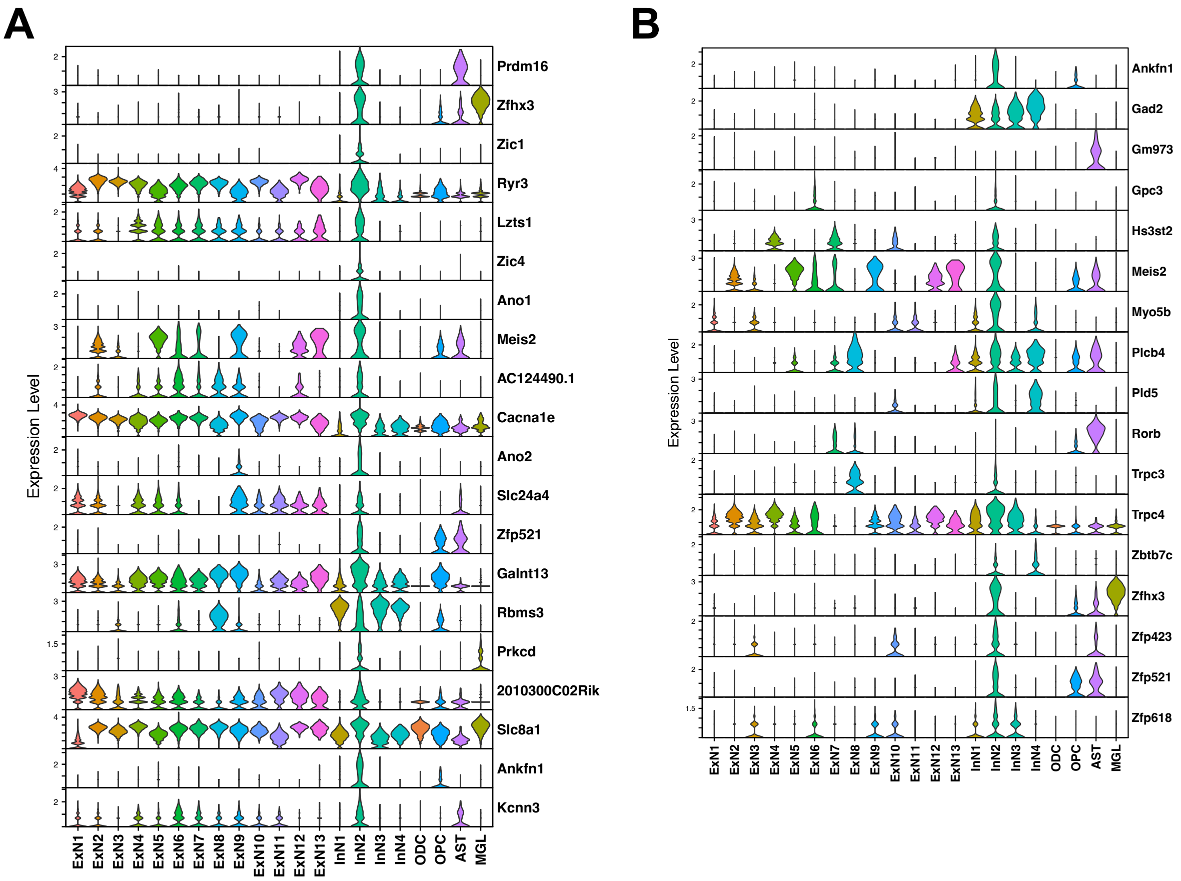

Supp. Fig. 4: A. Violin plots showing normalized expression of the top 20 differentially upregulated genes in the InN2 cluster, as compared to the other inhibitory neuron clusters. **B.** Violin plots showing normalized expression of the 17 genes comprising the Prdm16 regulon.

Supplementary figure 5

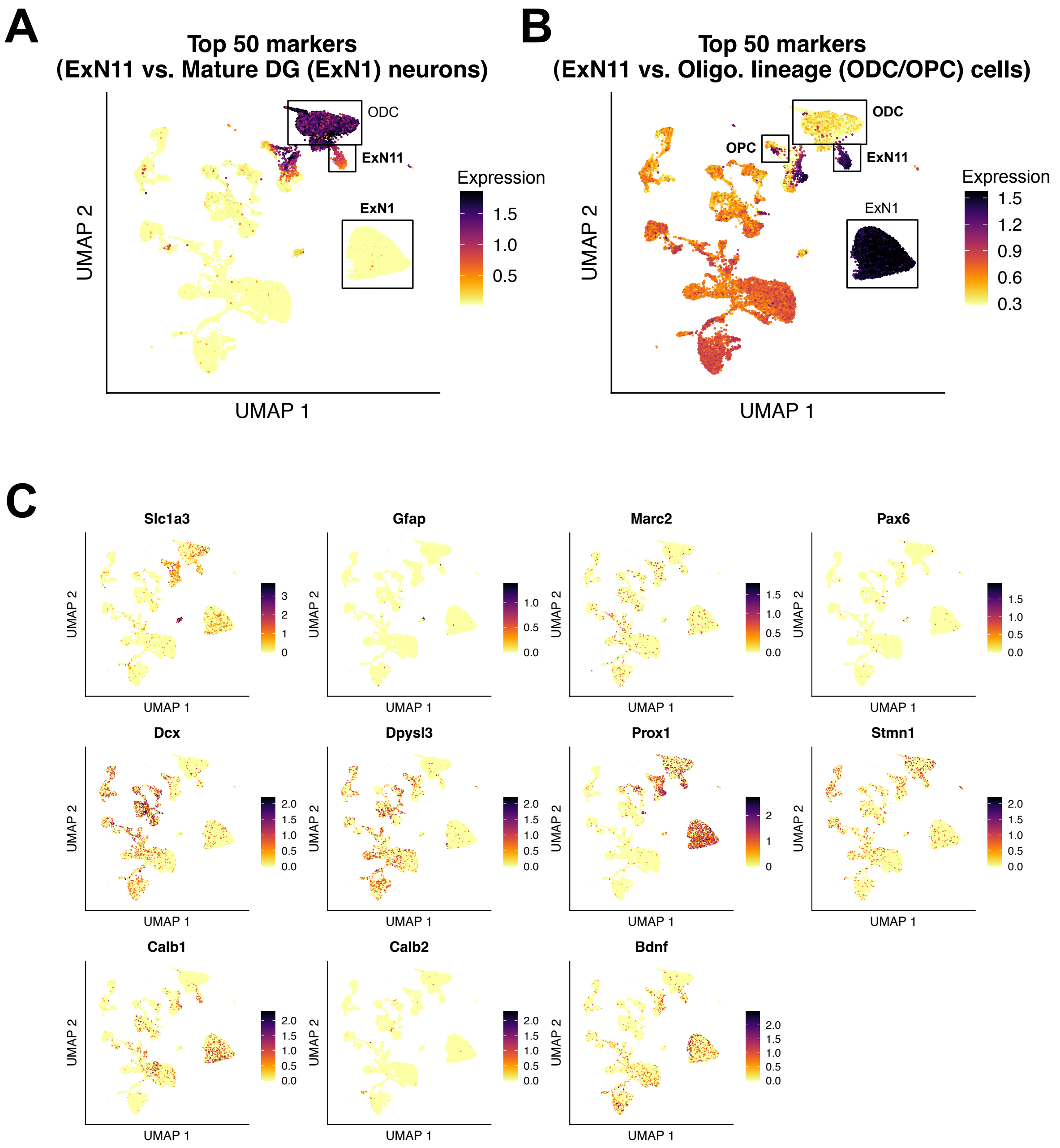

Supp. Fig. 5: A. UMAP plot depicting the average module score (expression) of the top 50 genes differentially expressed between the ExN11 cluster and the mature DG excitatory neuron cluster (ExN1). **B.** UMAP plot depicting the average module score (expression) of the top 50 genes differentially expressed between the ExN11 cluster and the oligodendrocyte and OPC clusters (ODC and OPC). **C.** UMAP plots depicting normalized expression of selected marker genes for radial glia/immature neurons/exercise-mediated neurogenesis.

Supplementary figure 6

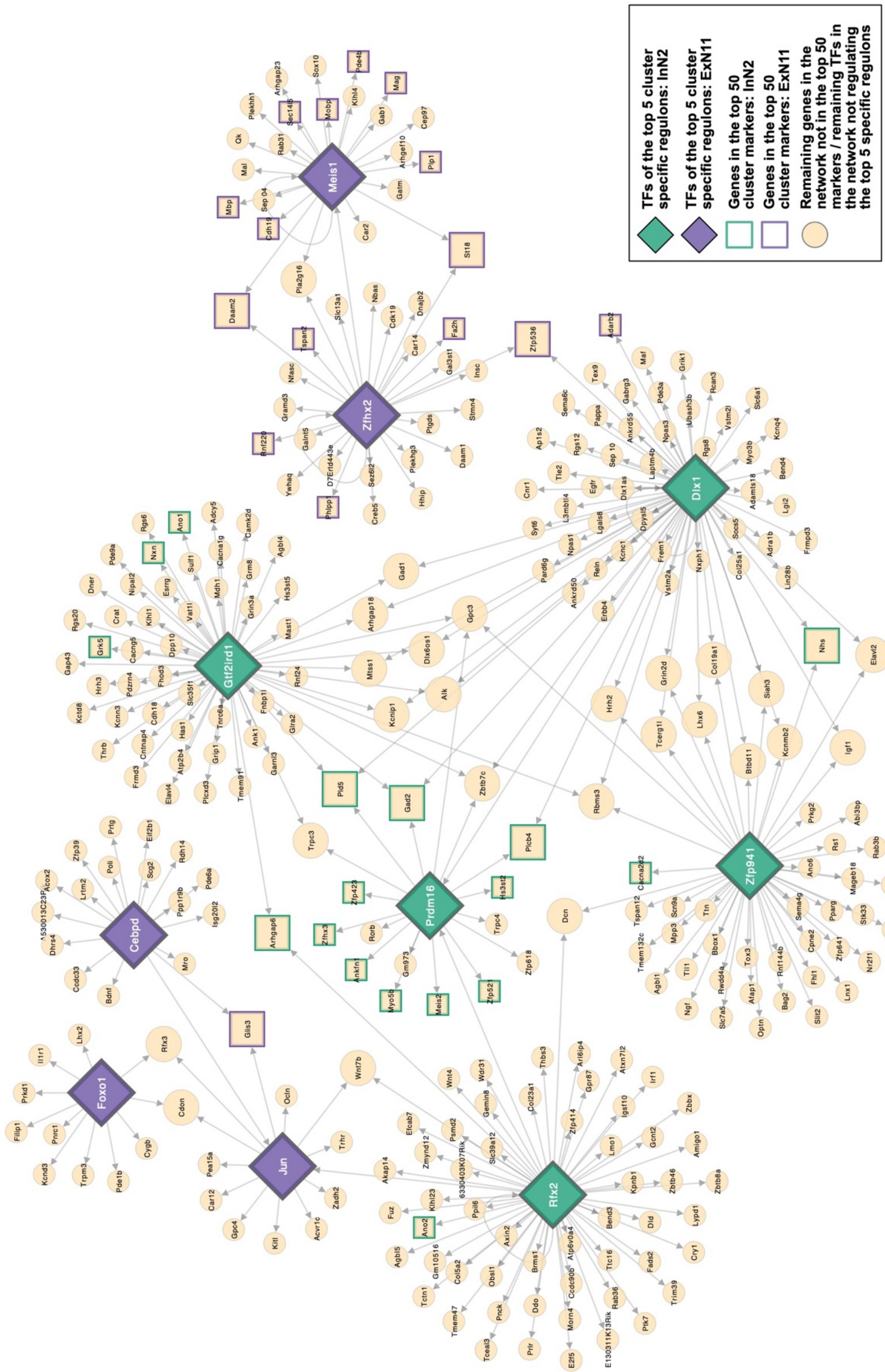

Supp. Fig. 6: Network plot with TFs and their respective regulon genes, specific to the InN2 and/or ExN11 cluster, indicating gene regulatory interactions between these two clusters (Larger nodes represent genes/TFs that connect two or more regulon networks).

Supplementary figure 7

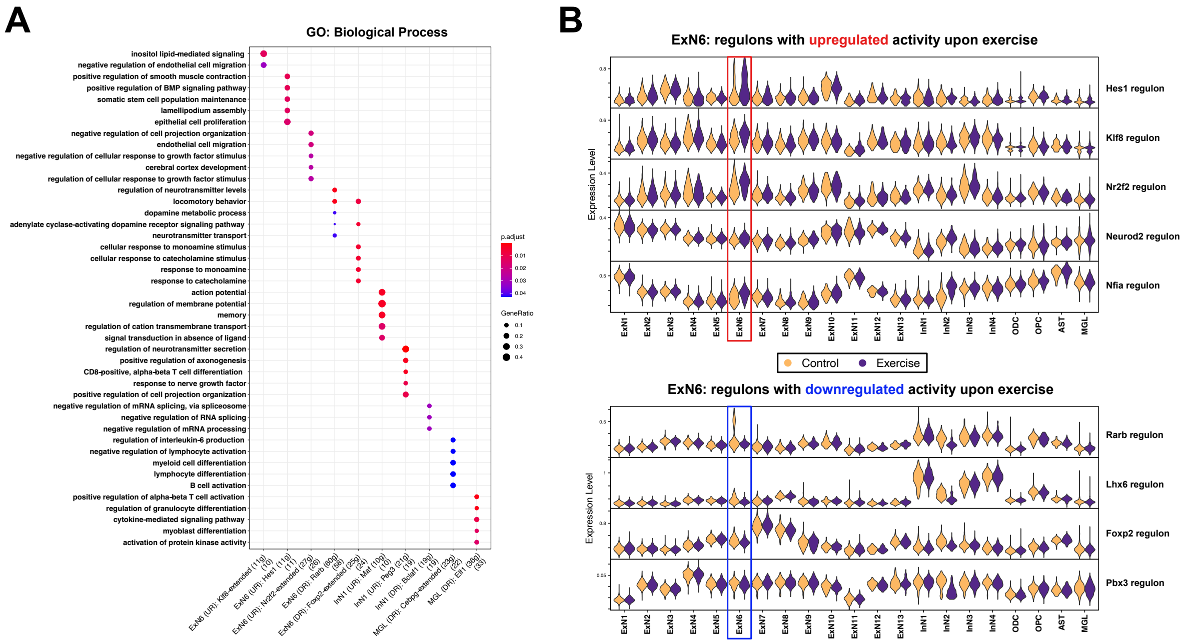

Supp. Fig. 7: A. Dotplot showing the top (up to 5) significant GO biological process terms enriched among genes making up the regulons with significant differential activity between cells from exercise and control conditions, in specific clusters as indicated in Fig. 4(C). **B.** Violin plots showing average module score (expression) of regulons with significant differential activity upon exercise, in the ExN6 cluster.

Supplementary figure 8

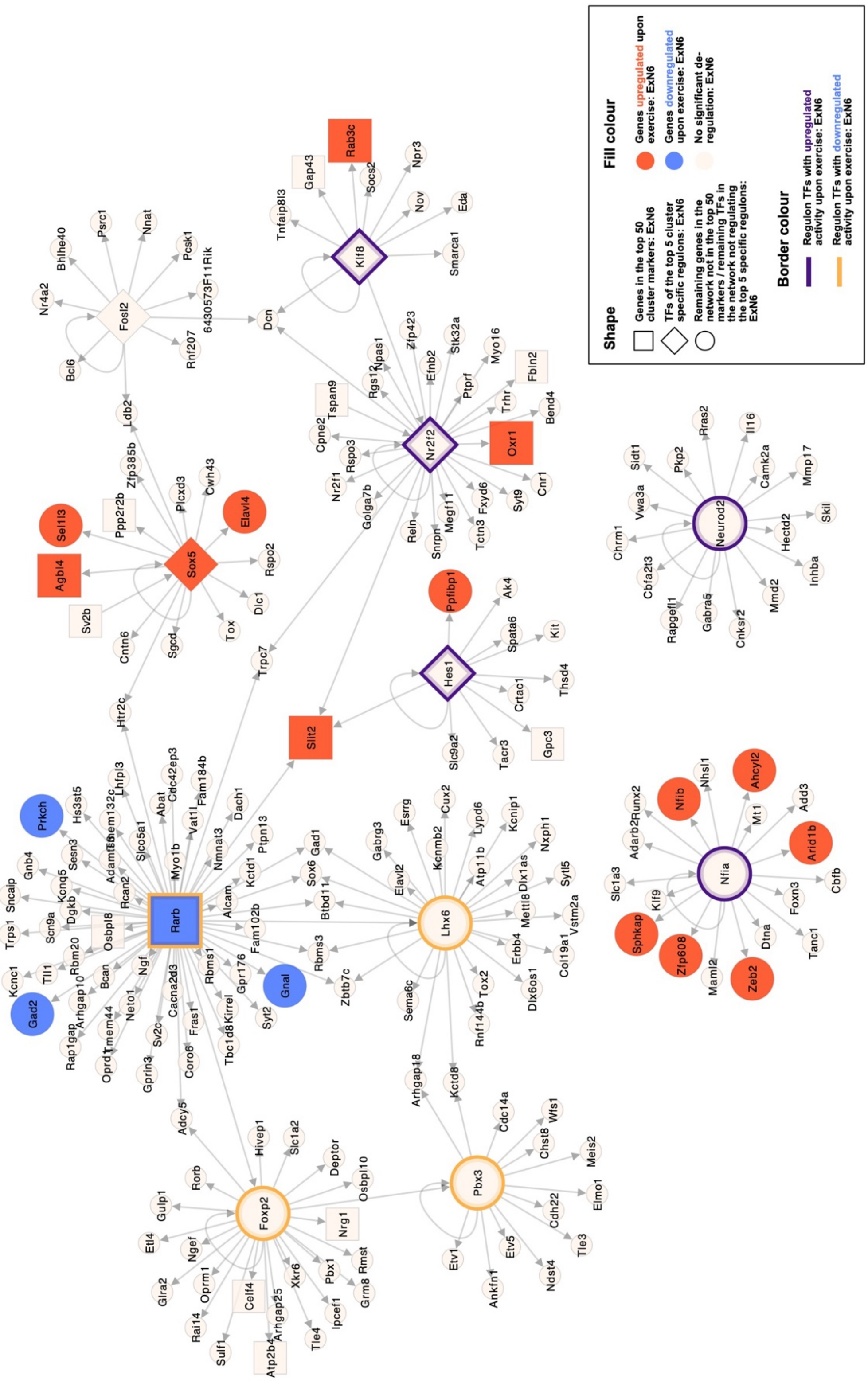

Supp. Fig. 8: Network plot with TFs and their respective regulon genes, specific to or showing differential activity in ExN6 upon exercise, indicating gene regulatory interactions in ExN6 cluster (Genes/TFs that are significantly deregulated upon exercise or are specifically overexpressed in ExN6 are represented bigger in size, while selected genes belonging to these regulons which are deregulated in the same direction as the regulon TF are colored in red or blue).
